# Post-neoadjuvant therapy lymph node response in patients with gastric adenocarcinoma

**DOI:** 10.1590/0102-672020260000049e1978

**Published:** 2026-10-09

**Authors:** Geraldo ISHAK, Luciana Roberta do Vale CORRÊA, Thaygo de Azevedo NEVES, Ricardo Ormanes MASSOUD, Ives Uchôa de AZEVEDO, Pedro Antônio Mufarrej HAGE, Williams Fernandes BARRA, Paulo Pimentel de ASSUMPÇÃO

**Affiliations:** 1 Universidade Federal do Pará, General and Digestive Surgery Service - Belém (PA), Brazil.; 2 Universidade do Estado do Pará, Center for Biological and Health Sciences - Belém (PA), Brazil.; 3 Universidade Federal do Pará, Medical Oncology Service - Belém (PA), Brazil.

**Keywords:** Stomach Neoplasms, Neoadjuvant Therapy, Drug Therapy, Neoplasm Staging, Neoplasias Gástricas, Terapia Neoadjuvante, Tratamento Farmacológico, Estadiamento de Neoplasias

## Abstract

**Background:**

Neoadjuvant chemotherapy is recommended as the standard of care for eligible patients with locally advanced resectable gastric adenocarcinoma. However, given the often-late diagnosis of this tumor, understanding post-neoadjuvant therapy lymph node involvement remains a determining factor in the prognosis of these patients.

**Aims:**

To evaluate the association between reduced tumor staging after neoadjuvant therapy and lymph node involvement in patients with gastric adenocarcinoma.

**Methods:**

This is a retrospective, analytical, observational study based on the medical records of patients who underwent combination therapy from 2018 to 2024. Pre-treatment clinical staging was performed according to institutional protocol, and the relationship between tumor and lymph node staging was established by comparing the initial clinical staging with the post-treatment pathological staging.

**Results:**

Eighty-nine patients were included in the analysis, with a reduction in tumor staging observed in 47.2% of cases, based on pathological T-cell analysis. The mean number of affected lymph nodes was significantly higher in non-responder patients compared to responders (5.0±6.3 versus 1.1±2.2; p<0.001). Simple logistic regression analysis identified that the number of affected lymph nodes was associated with the absence of tumor response in univariate analysis, presenting an odds ratio of 1.317 (95%CI 1.099-1.579; p=0.003).

**Conclusions:**

The absence of reduction in tumor staging was associated with a higher residual lymph node burden, highlighting the prognostic relevance of lymph node involvement in post-therapeutic evaluation.

## ARTICLE HIGHLIGHTS

• Primary tumor downstaging was achieved in 47.2% of patients following neoadjuvant chemotherapy.

• Non-responders presented a significantly higher residual nodal burden than responders (a mean of 5.0 vs. 1.1 metastatic lymph nodes).

• Each additional metastatic lymph node increased the odds of treatment failure by 31.7% (OR=1.317).

• Persistent lymph node metastases after neoadjuvant therapy may represent a marker of aggressive tumor biology and poorer prognosis.

## Summary

**CENTRAL MESSAGE:**

Gastric cancer management requires a multidisciplinary and individualized approach tailored to the characteristics of each patient and institution. Besides surgical treatment, perioperative chemotherapy has become an essential component of modern management for locally advanced disease. Because lymph nodes are common sites of metastatic spread, residual nodal disease after neoadjuvant therapy represents an important marker of treatment response and long-term prognosis.

**PERSPECTIVES:**

This study demonstrated that the absence of primary tumor downstaging after neoadjuvant chemotherapy was associated with a greater residual nodal burden, reflected by a higher number of metastatic lymph nodes in the surgical specimen. These findings are consistent with the well-established prognostic importance of lymph node status following neoadjuvant treatment for gastric cancer and suggest that persistent nodal disease may indicate a more aggressive tumor biology and reduced sensitivity to systemic therapy. Quantitative assessment of metastatic lymph nodes may therefore provide complementary prognostic information and contribute to subsequent therapeutic decision-making.

## VISUAL ABSTRACT

**Figure:**
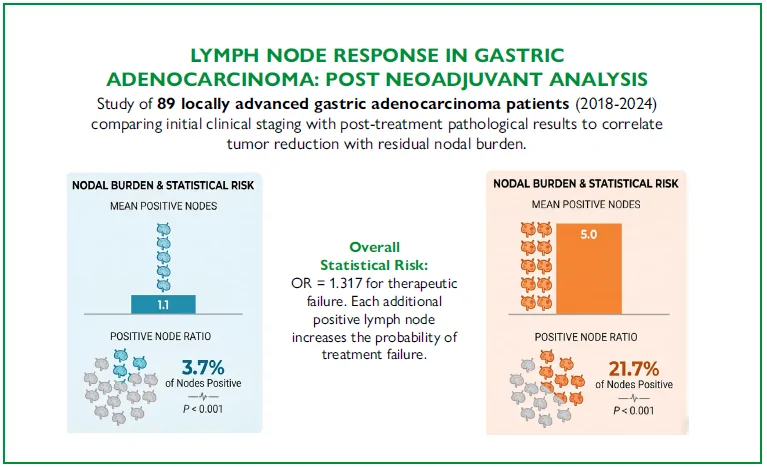
VISUAL ABSTRACT

## RESUMO

**Racional:**

A quimioterapia neoadjuvante é recomendada como padrão de tratamento em pacientes elegíveis com adenocarcinoma gástrico ressecável localmente avançado. Entretanto, tendo em vista o diagnóstico muitas vezes tardio desse tumor, compreender o acometimento linfonodal pós-neoadjuvância permanece como fator determinante no prognóstico desses pacientes.

**Objetivos:**

Avaliar a associação entre redução do estadiamento tumoral após neoadjuvância e acometimento linfonodal em pacientes com adenocarcinoma gástrico.

**Métodos:**

Trata-se de um estudo observacional analítico retrospectivo, baseado nos prontuários dos pacientes com submetidos à terapia combinada no período entre 2018 e 2024. O estadiamento clínico pré-tratamento foi realizado conforme protocolo institucional, e a relação entre estadiamento tumoral e linfonodal foi estabelecida por meio da comparação entre o estadiamento clínico inicial e o patológico pós-tratamento.

**Resultados:**

Foram incluídos 89 pacientes na análise, observando-se redução do estadiamento tumoral em 47,2% dos casos, baseando-se na análise do T patológico. Verificou-se que o número médio de linfonodos acometidos foi significativamente maior entre os pacientes não respondedores quando comparados aos respondedores (5,0±6,3 versus 1,1±2,2; p<0,001). Na análise por regressão logística simples, identificou-se que o número de linfonodos acometidos associou-se à ausência de resposta tumoral em análise univariada, apresentando razão de chances (OR) de 1,317 (IC95% 1,099-1,579; p=0,003).

**Conclusões:**

A ausência de redução do estadiamento tumoral esteve associada a uma maior carga linfonodal residual, evidenciando a relevância prognóstica do comprometimento linfonodal na avaliação pós-terapêutica.

## INTRODUCTION

Gastric cancer is a multifactorial disease that commonly develops through the inflammation-metaplasia-dysplasia-carcinoma sequence described in the gastric precancerous cascade^9^. It is currently the fifth most frequently diagnosed malignancy worldwide and the fourth leading cause of cancer-related mortality, excluding non-melanoma skin cancer^33^. In Brazil, more than 13,000 new cases were estimated for the 2023-2025 triennium, and gastric cancer represents the second most incident malignancy in the Northern region of the country^30^.

Tumor staging is based on the Tumor, Node, and Metastasis (TNM) classification system, in which T describes the depth of tumor invasion, N indicates the presence and extent of regional lymph node metastasis, and M reflects the presence of distant metastatic disease^3^. Recent evidence evaluating the updated staging criteria in patients undergoing multimodal treatment has demonstrated that TNM classification remains a reasonable independent predictor of survival. Nevertheless, patients classified as T0N0 or T0N+ after treatment may still experience unfavorable outcomes, highlighting the prognostic importance of residual lymph node involvement despite adequate local tumor control^15^.

The management of gastric cancer is inherently multidisciplinary and should be individualized according to patient characteristics, institutional expertise, and available therapeutic resources. Currently, early gastric cancer is defined as a lesion confined to the mucosa or submucosa, regardless of lymph node status, without invasion of the muscularis propria^14^. This distinction is clinically relevant because current international guidelines recommend endoscopic resection for selected T1aN0M0 tumors, achieving excellent oncological outcomes^7^ ^,^ ^16^ ^,^ ^22^.

For more advanced lesions or tumors unsuitable for endoscopic treatment, surgical resection remains the cornerstone of curative therapy. Depending on tumor location, distal tumors are generally managed with subtotal gastrectomy, whereas proximal tumors require total gastrectomy^7^.

Regional lymph node metastases are among the most important prognostic determinants in gastric cancer and represent the most frequent sites of disease dissemination^12^. Previous studies have demonstrated that adequate pathological staging requires the retrieval of at least 16 lymph nodes, a target that is consistently achieved with D2 lymphadenectomy^6^. Accordingly, current international guidelines recommend D2 lymphadenectomy for all tumors staged as T2 or higher, as well as for node-positive disease, regardless of whether subtotal or total gastrectomy is performed^13^.

Beyond surgery, perioperative chemotherapy has become an essential component of treatment for locally advanced gastric cancer. This therapeutic strategy combines preoperative chemotherapy with curative surgical resection followed by postoperative chemotherapy to maximize the likelihood of achieving a pathological complete response^38^.

Recent studies have demonstrated that multimodal treatment significantly increases the probability of pathological complete response compared with surgery followed by postoperative chemotherapy alone. Among pretreatment factors, higher tumor differentiation appears to be the strongest predictor of complete pathological response^7^.

Furthermore, a recent meta-analysis including 13 studies evaluating neoadjuvant therapy for resectable gastric cancer demonstrated higher pathological complete response rates with neoadjuvant treatment, although no significant differences were observed in overall survival or disease-free survival^21^. In this sense, the MAGIC trial first established the survival benefit of perioperative chemotherapy compared with surgery alone. Subsequently, the FLOT4 trial demonstrated the superiority of the FLOT (Fluorouracil, Leucovorin, Oxaliplatin, and Docetaxel) regimen over epirubicin-based protocols, consolidating FLOT as the current standard treatment for patients with locally advanced resectable gastric adenocarcinoma^1^ ^,^ ^2^ ^,^ ^10^ ^,^ ^22^ ^,^ ^26^ ^,^ ^32^.

Given this scenario, further investigation of pathological response markers-particularly residual lymph node involvement-and their relationship with primary tumor downstaging is warranted. Therefore, the present study aimed to evaluate the association between primary tumor downstaging after neoadjuvant therapy and residual lymph node involvement in patients with gastric adenocarcinoma treated at a tertiary referral center in Northern Brazil.

## METHODS

This retrospective, analytical, cross-sectional observational study was based on data extracted from the medical records of patients with gastric adenocarcinoma who underwent neoadjuvant chemotherapy followed by surgical resection at the João de Barros Barreto University Hospital (HUJBB), Belém, Pará, Brazil. The study was conducted in accordance with the ethical principles established by the Brazilian National Health Council Resolution No. 466/2012 and was approved by the Institutional Research Ethics Committee on June 27, 2025 (Approval No. 7,674,593). The confidentiality of patient information was ensured throughout the study, and all data were anonymized before analysis.

Patients with histologically confirmed gastric adenocarcinoma who underwent neoadjuvant chemotherapy followed by surgical resection with lymphadenectomy were eligible for inclusion. The pathological report was required to include the total number of resected lymph nodes and the number of metastatic lymph nodes, in addition to sufficient information to determine both the pretreatment clinical T stage (cT) and the post-treatment pathological T stage (ypT). Patients were excluded if the information required to compare cT and ypT was unavailable or if the pathological reports lacked the variables necessary for the primary analyses.

All patients received four cycles of the FLOT regimen over approximately eight weeks according to the institutional protocol. After completion of neoadjuvant chemotherapy, patients were referred back to the Digestive Surgery team and underwent surgical resection approximately 60 days after treatment completion.

Pretreatment clinical staging was performed according to the institutional standard protocol and included computed tomography of the chest and abdomen, upper gastrointestinal endoscopy, and diagnostic laparoscopy to determine the locoregional and systemic extent of disease before the initiation of neoadjuvant therapy. Clinical TNM staging was established based on the combined findings of endoscopy and computed tomography.

Tumor response was defined as primary tumor downstaging, determined by comparing the pretreatment clinical T stage (cT) with the post-treatment pathological T stage (ypT). Patients were considered responders when a reduction in T stage was observed after neoadjuvant therapy, whereas those without a reduction in pathological T stage were classified as non-responders.

Demographic and clinical variables included age, sex, smoking status, and Charlson Comorbidity Index (CCI). Clinicopathological and surgical variables included tumor location, ypT, ypN, and ypM stages, Lauren histological classification, type of surgical procedure, total number of resected lymph nodes, and number of metastatic lymph nodes, the latter being considered the residual nodal burden.

Statistical analyses were performed using the Statistical Package for the Social Sciences (SPSS), version 20.0^24^. Categorical variables were expressed as absolute and relative frequencies with corresponding 95% confidence intervals (95% CIs), whereas continuous variables were presented as mean±standard deviation (SD) or median with interquartile range (25th-75th percentiles), according to data distribution. Comparisons between responders and non-responders were performed using Pearson’s χ^2^ test for categorical variables and the Mann-Whitney U test for continuous variables because of the non-normal distribution of the data. The association between the number of metastatic lymph nodes and response to neoadjuvant treatment was evaluated using univariate logistic regression, with results expressed as odds ratios (ORs) and corresponding 95%CI. A two-sided p-value<0.05 was considered statistically significant^18^.

## RESULTS

A total of 89 patients with gastric cancer who underwent neoadjuvant treatment were included in the study. Most patients were male (61.8%), with a mean age of 58.3±12.7 years and a median age of 59.0 years. Age distribution was similar between patients younger than 60 years (52.8%) and those aged 60 years or older (47.2%). A history of smoking was reported by 41.6% of patients, whereas 51.7% were non-smokers. The mean Charlson Comorbidity Index was 3.6±1.2, indicating a substantial burden of comorbidities in the study population (Table 1).

**Table 1.** Clinical and demographic characteristics of patients with gastric cancer undergoing neoadjuvant treatment.

Variables	Frequency (n=89)	Percentage (%)	95%CI
Sex			
Male	55	61.8	51.7-70.8
Female	34	38.2	29.2-48.3
Age (years)			
Mean (SD)	58.3 (12.7)		55.8-60.6
Median (p25-p75)	59 (57-69)		54-63
Age Group (years)			
< 60	47	52.8	41.6-64.0
≥ 60	42	47.2	36.0-58.4
Smoker			
Yes	37	41.6	31.5-51.7
No	46	51.7	41.6-62.9
Not Applicable	6	6.7	2.2-12.4
Charlson’s Index			
Mean (SD)	3.6 (1.2)		3.3-3.8
Median (p25-p75)	3.0 (3.0-4.5)		4.0-4.0

SD: standard deviation; p: percentile; 95%CI: 95% confidence interval.

Concerning primary tumor staging, T3 tumors were the most frequent (48.3%), followed by T2 (25.8%) and T4 tumors (24.7%). Regarding regional lymph node status, nearly half of the patients were classified as N0 (47.2%), whereas 32.6% and 18.0% were classified as N1 and N2, respectively. Almost all patients had no evidence of distant metastasis (M0, 98.9%). According to Lauren’s histological classification, the intestinal subtype was the most prevalent (46.1%), followed by the diffuse (32.6%) and mixed (7.9%) subtypes. Histological subtype was not reported in 13.5% of cases (Table 2).

**Table 2.** Tumor, Node, and Metastasis staging and Lauren’s histological classification of patients with gastric cancer undergoing neoadjuvant treatment.

Variables	Frequency (n=89)	Percentage (%)	95%CI
Primary Tumor Staging			
T2	23	25.8	17.0-35.2
T3	43	48.3	38.6-59.1
T4	22	24.7	15.9-34.1
Not Applicable	1	1.1	0.0-3.4
Regional Lymph Nodes			
N0	42	47.2	37.5-58.0
N1	29	32.6	23.9-42.0
N2	16	18	11.4-26.1
N3	1	1.1	0.0-3.4
Not Applicable	1	1.1	0.0-3.4
Distant Metastases			
M0	88	98.9	96.0-100.0
Not Applicable	1	1.1	0.0-3.4
Lauren’s Type			
Diffuse	29	32.6	23.6-43.8
Intestinal	41	46.1	36.0-56.2
Mixed	7	7.9	3.4-13.5
Not Applicable	12	13.5	5.6-21.3

95%CI: 95% confidence interval.

Total gastrectomy was the most frequently performed surgical procedure (50.6%), followed by subtotal gastrectomy (48.3%). The mean number of resected lymph nodes was 22.9±12.1, with a median of 20 lymph nodes. The mean number of metastatic lymph nodes was 3.2±5.2, with a median of one metastatic lymph node. Primary tumor downstaging following neoadjuvant therapy was observed in 47.2% of patients (Table 3).

**Table 3.** Surgical characteristics, lymph node findings, and treatment response.

Variables	Frequency (n=89)	Percentage (%)	95%CI
Type of Surgery			
Total Gastrectomy	45	50.6	39.3-60.7
Subtotal Gastrectomy	43	48.3	38.2-59.5
Subtotal Esophagectomy+ Proximal Gastrectomy	1	1.1	0.0-3.4
Total Lymph Nodes			
Mean (SD)	22.9 (12.1)		20.5-25.7
Median (p25-p75)	20.0 (14.0-29.0)		17.0-24.5
Metastatic Lymph Nodes			
Mean (SD)	3.2 (5.2)		2.2-4.3
Median (p25-p75)	1.0 (0.0-4.0)		0.0-2.0
Response to Treatment			
Primary Tumor Shrinkage	42	47.2	36.0-58.4

SD: standard deviation; p: percentile; 95%CI: 95% confidence interval.

No statistically significant differences were observed between responders and non-responders regarding sex (p=0.126), mean age (p=0.175), age group (p=1.000), smoking status (p=1.000), or Charlson Comorbidity Index (p=0.374), indicating comparable clinical and demographic characteristics between the two groups (Table 4).

**Table 4.** Comparison of clinical and demographic characteristics according to response to neoadjuvant treatment in patients with gastric cancer.

Variables	Response to Treatment		p-value
	Yes (n=42)	No (n=47)	
Sex			
Male, n (%)	22 (52.4)	33 (70.2)	0.126^a^
Female, n (%)	20 (47.6)	14 (29.8)	
Age (years)			
Mean (SD)	58.4 (11.8)	58.3 (13.6)	0.175^b^
Median (p25-p75)	59.0 (51.0-67.0)	57.0 (46.5-69.0)	
Age Group (years)			
<60, n (%)	22 (52.4)	25 (53.2)	1.000^a^
≥60, n (%)	20 (47.6)	22 (46.8)	
Smoker			
Yes, n (%)	17 (40.5)	20 (42.6)	1.000^a^
No, n (%)	22 (52.4)	24 (51.1)	
Not Applicable, n( %)	3 (7.1)	3 (6.4)	
Charlson’s Index			
Mean (SD)	3.5 (1.2)	3.6 (1.3)	0.374^b^
Median (p25-p75)	3.0 (3.0-4.0)	3.0 (2.5-5.0)	

SD: standard deviation; p: percentile; ^a^χ^2^ Test; ^b^Mann-Whitney Test.

Comparison of tumor staging between responders and non-responders showed no statistically significant association for either primary tumor stage (p=0.228) or regional lymph node stage (p=0.939). Likewise, no significant difference was observed in Lauren’s histological classification between the groups (p=0.942) (Table 5).

**Table 5.** Tumor, Node, and Metastasis staging and Lauren’s histological classification according to treatment response.

Variables	Response to Treatment		p-value
	Yes (n=42)	No (n=47)	
Primary			
Tumor Staging N (%)			
T2	10 (23.8)	13 (27.7)	0.228^a^
T3	18 (42.9)	25 (53.2)	
T4	14 (33.3)	8 (17.0)	
Not Applicable	0 (0.0)	1 (2.1)	
Regional Lymph Nodes N (%)			
N0	20 (47.6)	22 (46.8)	0.939^a^
N1	15 (35.7)	14 (29.8)	
N2	7 (16.7)	9 (19.1)	
N3	0 (0.0)	1 (2.1)	
Not Applicable	0 (0.0)	1 (2.1)	
Lauren’s Type N (%)			
Diffuse	11 (26.2)	18 (38.2)	0.942^a^
Intestinal	17 (40.5)	24 (51.1)	
Mixed	3 (7.1)	4 (8.5)	
Not Applicable	11 (26.2)	1 (2.1)	

^a^χ^2^ Test.

A trend toward a higher frequency of subtotal gastrectomy was observed among responders, although this difference did not reach statistical significance (p=0.071). The total number of resected lymph nodes was comparable between the two groups (p=0.664). In contrast, the number of metastatic lymph nodes was significantly lower among responders than among non-responders, both in terms of mean values (1.1±2.2 vs. 5.0±6.3) and median values (0 vs. 2), with a highly significant difference (p<0.001) (Table 6).

**Table 6.** Comparison of surgical and lymph node characteristics according to response to neoadjuvant therapy.

Variables	Response to Treatment		p-value
	Yes (n=42)	No (n=47)	
Type of Surgery			
Total Gastrectomy	17 (40.5)	28 (59.6)	0.071^a^
Subtotal Gastrectomy	25 (59.5)	18 (38.3)	
Subtotal Esophagectomy+ Proximal Gastrectomy	0 (0.0)	1 (1.2)	
Total Lymph Nodes			
Mean (SD)	22.7 (12.9)	23.2 (11.3)	0.664^b^
Median (p25-p75)	21 (14-30)	20 (14-27)	
Metastatic Lymph Nodes			
Mean (SD)	1.1 (2.2)	5.0 (6.3)	<0.001^b^
Median (p25-p75)	0 (0-1)	2 (1-8)	

SD: standard deviation; p: percentile; ^a^χ^2^ Test; ^b^Mann-Whitney Test.

Univariate logistic regression analysis demonstrated a significant association between the number of metastatic lymph nodes and response to neoadjuvant treatment. Each additional metastatic lymph node was associated with a higher likelihood of failure to achieve primary tumor downstaging (OR=1.317; 95%CI 1.099-1.579; p=0.003) (Table 7).

**Table 7.** Association between the number of metastatic lymph nodes and response to neoadjuvant treatment.

Variables	Response to Treatment		p-value	OR (95%CI)
	Yes (n=42)	No (n=47)		
Metastatic Lymph Nodes				
Mean (SD)	1.1 (2.2)	5.0 (6.3)	0.003	1.317 (1.099-1.579)
Median (p25-p75)	0 (0-1)	2 (1-8)		

SD: standard deviation; p: percentile; OR: odds ratio; 95%CI: confidence interval; ^a^χ^2^ Test.

A total of 2,045 resected lymph nodes were evaluated, of which 272 (13.3%) harbored metastatic disease. Among responders, only 35 lymph nodes (3.7%) were metastatic, whereas 237 lymph nodes (21.7%) were metastatic in non-responders. This difference was statistically significant (p<0.001), demonstrating a clear association between residual lymph node involvement and failure to respond to neoadjuvant therapy. Accordingly, metastatic lymph nodes were substantially more frequent among patients who did not achieve primary tumor downstaging (Table 8).

**Table 8.** Association between metastatic lymph node involvement relative to the total number of resected lymph nodes and response to neoadjuvant treatment.

Total Lymph Nodes (n=2045)	Response to Treatment		p-value
	Yes (n=954)	No (n=1,091)	
Metastatic Lymph Nodes (n=272)			
Yes N (%)	35 (3.7)	237 (21.7)	<0.001^a^
No N (%)	919 (96.3)	854 (78.3)	

^a^χ^2^ Test.

## DISCUSSION

Perioperative or neoadjuvant chemotherapy is currently recommended by major international guidelines and supported by robust clinical evidence, particularly the FLOT regimen for eligible patients. Nevertheless, therapeutic response remains heterogeneous, and the persistence of residual nodal disease may reflect both intrinsic tumor resistance and a greater initial tumor burden. These concepts are consistent with the findings of the present study, in which failure to achieve primary tumor downstaging after neoadjuvant therapy was associated with a greater residual nodal burden, reinforcing lymph node involvement as one of the main prognostic determinants in gastric cancer^1^ ^,^ ^2^ ^,^ ^10^ ^,^ ^16^ ^,^ ^22^ ^,^ ^26^ ^,^ ^27^ ^,^ ^29^ ^,^ ^32^.

The dissociation between regression of the primary tumor and persistence of nodal disease has important clinical implications. Previous studies evaluating regression in metastatic lymph nodes and the prognostic impact of post-treatment nodal status have demonstrated that assessment of the lymph node compartment provides additional prognostic information and plays a central role in postoperative risk stratification. These findings are consistent with those observed in the present study^4^ ^,^ ^19^ ^,^ ^29^ ^,^ ^32^ ^,^ ^34^.

Although categorical nodal staging (ypN) did not differ significantly between responders and non-responders, the absolute number of metastatic lymph nodes was significantly different, suggesting that quantitative differences within the same ypN category may carry important clinical implications. Recent studies have also demonstrated that the lymph node ratio represents an independent prognostic factor in patients undergoing neoadjuvant therapy^23^ ^,^ ^28^ ^,^ ^37^.

Accurate interpretation of residual nodal burden depends on adequate oncologic lymphadenectomy and meticulous pathological examination. International recommendations and accumulated evidence indicate that an adequate number of lymph nodes must be retrieved to minimize stage migration. In the present study, the mean number of resected lymph nodes exceeded the recommended minimum threshold, thereby strengthening the reliability of the pathological staging and the validity of our findings^5^ ^,^ ^6^ ^,^ ^8^ ^,^ ^18^ ^,^ ^31^ ^,^ ^36^.

Despite advances in multimodal treatment, therapeutic response may differ between the primary tumor and the lymph node compartment. Pathological evaluation following neoadjuvant therapy has increasingly been used to estimate tumor sensitivity and residual disease burden. Long-term analyses of the MAGIC trial suggest that post-treatment nodal status may be an even stronger prognostic indicator than regression of the primary tumor itself^26^ ^,^ ^32^.

Regarding pretreatment staging, the absence of routine endoscopic ultrasonography may have contributed to inaccuracies in clinical T staging. Previous meta-analyses have demonstrated that the diagnostic performance of endoscopic ultrasonography and computed tomography varies according to tumor substage and clinical context. Therefore, defining tumor response exclusively by comparing clinical T stage (cT) with pathological T stage (ypT) should be interpreted with caution and represents an inherent methodological limitation of the present study^20^ ^,^ ^25^ ^,^ ^35^.

Although the institutional database did not systematically include tumor regression grading (TRG), validated grading systems, such as the Becker classification and contemporary reviews describing the histopathological features of tumor regression after neoadjuvant therapy, are well established in the literature. Incorporating standardized TRG assessment into institutional practice may improve pathological evaluation and should be considered in future investigations^7^ ^,^ ^11^ ^,^ ^17^.

The main limitations of this study include its retrospective design and the reliance on pretreatment clinical staging to define primary tumor downstaging. Consequently, the present findings should be interpreted as observational associations and do not allow causal inferences.

## CONCLUSIONS

The lack of primary tumor reduction following neoadjuvant chemotherapy was associated with a higher residual lymph node burden, evidenced by a greater number of involved lymph nodes in the surgical specimen. This result aligns with the prognostic significance widely attributed to the lymph nodes, in the post-neoadjuvant assessment of gastric cancer. It reinforces the notion that persistent lymph node disease may reflect more aggressive tumor biology and reduced sensitivity to systemic treatment, highlighting the importance of the quantitative analysis of involved lymph nodes as a complementary tool for prognostic stratification and subsequent treatment planning. However, given the retrospective design and methodological limitations inherent to clinical staging and the available variables, these results should be interpreted as observational associations, underscoring the need for further research to better understand the clinical impact of these relationships.

## Data Availability

The datasets generated and/or analyzed during the current study are available from the corresponding author upon reasonable request.
